# Proliferation of a bloom-forming phytoplankton via uptake of polyphosphate-accumulating bacteria under phosphate-limiting conditions

**DOI:** 10.1093/ismeco/ycaf192

**Published:** 2025-12-05

**Authors:** Seiya Fukuyama, Fumiko Usami, Ryuichi Hirota, Ayano Satoh, Shizuka Ohara, Ken Kondo, Yuki Gomibuchi, Takuo Yasunaga, Toshimitsu Onduka, Akio Kuroda, Kazuhiko Koike, Shoko Ueki

**Affiliations:** Institute of Plant Science and Resources, Okayama University, Chuo 2-20-1, Kurashiki City, Okayama 710-0046, Japan; Nagoya Seiraku Co., Ltd., 310 Nakasuna-cho, Tempaku-ku, Nagoya, Aichi 468-8588, Japan; Institute of Plant Science and Resources, Okayama University, Chuo 2-20-1, Kurashiki City, Okayama 710-0046, Japan; Graduate School of Integrated Sciences for Life, Hiroshima University, 1-4-4 Kagamiyama, Higashi-Hiroshima, Hiroshima 739-8528, Japan; Graduate School of Interdisciplinary Science and Engineering in Health Systems, Okayama University, 3-1-1 Tsushima-naka, Kita-ku, Okayama-shi 700-8530, Japan; Graduate School of Integrated Sciences for Life, Hiroshima University, 1-4-4 Kagamiyama, Higashi-Hiroshima, Hiroshima 739-8528, Japan; Hatsukaichi Branch, Fisheries Technology Institute, Fisheries Research and Education Agency, 2-17-5 Maruishi, Hatsukaichi, Hiroshima 739-0452, Japan; Research Institute of Environment, Agriculture and Fisheries, Osaka Prefecture, 442 Shakudo, Habikino City, Osaka 583-0862, Japan; Department of Physics and Information Technology, Faculty of Computer Science and Systems Engineering, Kyushu Institute of Technology, 680-4 Kawazu, Iizuka, Fukuoka 820-8502, Japan; Department of Physics and Information Technology, Faculty of Computer Science and Systems Engineering, Kyushu Institute of Technology, 680-4 Kawazu, Iizuka, Fukuoka 820-8502, Japan; Hatsukaichi Branch, Fisheries Technology Institute, Fisheries Research and Education Agency, 2-17-5 Maruishi, Hatsukaichi, Hiroshima 739-0452, Japan; Graduate School of Integrated Sciences for Life, Hiroshima University, 1-4-4 Kagamiyama, Higashi-Hiroshima, Hiroshima 739-8528, Japan; Graduate School of Integrated Sciences for Life, Hiroshima University, 1-4-4 Kagamiyama, Higashi-Hiroshima, Hiroshima 739-8528, Japan; Institute of Plant Science and Resources, Okayama University, Chuo 2-20-1, Kurashiki City, Okayama 710-0046, Japan

**Keywords:** bacterivory, mixotrophy, *Heterosigma akashiwo*, bloom-forming algae, phosphate, polyphosphate

## Abstract

Harmful algal blooms negatively impact the ecosystem and fisheries in affected areas. Eutrophication is a major factor contributing to bloom occurrence, and phosphorus is particularly important in limiting the growth of bloom-forming algae. Although algae efficiently utilize orthophosphate (Pi) as a phosphorous source over other molecular forms, Pi is often limited in the marine environment. While uptake and utilization of soluble inorganic and organic phosphorous by bloom-forming algae has been extensively studied, the details of geochemical and biological phosphorous cycling remain to be elucidated. Here, we report for the first time that the bloom-forming alga *Heterosigma akashiwo* can phagocytose bacteria and grow under phosphate-depleted conditions. The addition of *Vibrio comitans* to Pi-depleted *H. akashiwo* enabled the alga propagate to high cell densities, whereas other bacterial strains had only a minor effect. Importantly, *V. comitans* accumulates polyphosphate—a linear polymer of Pi—at high levels. The extent of algal proliferation induced by the addition of *Vibrio* species and polyphosphate-accumulating *Escherichia coli* correlated strongly with their polyphosphate content, indicating that bacterial polyphosphate served as an alternative PO_4_^3−^ source for *H. akashiwo*. The direct uptake of polyphosphate-accumulating bacteria through algal phagocytosis may represent a novel biological phosphorous-cycling pathway in marine ecosystems. The role of polyphosphate-accumulating marine bacteria as a hidden phosphorous source required for bloom formation warrants further investigation.

## Graphical Abstract

**Graphical Abstract:**
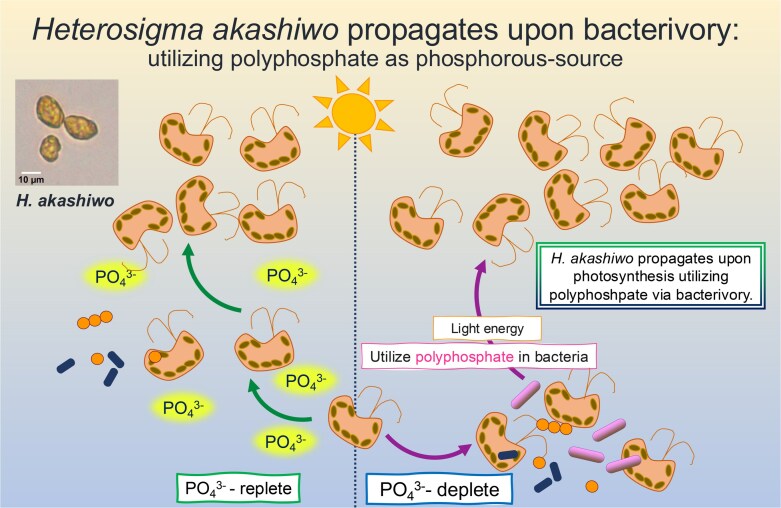

## Introduction

Phytoplankton are primary producers, and their photosynthetic output supports the entire aquatic food web [1–4]. Phytoplankton propagation is limited by various factors, including temperature, light intensities, salinity, and nutrient availability [5–10]. Among the diverse phytoplankton species, those that occasionally propagate to form high-density bloom and exert negative effects on ecosystems are collectively termed harmful algal bloom (HAB) species [6–8, 11]. These bloom-forming species are characterized by their competitiveness over other phytoplankton, typically due to their ability to utilize nutrients in diverse chemical forms, their efficient uptake mechanisms [12–17], and their allelopathic effects against other species [18, 19].

Among the various nutrients, nitrogen (N), iron (Fe), and phosphorus (P) are macronutrients that limit algal growth and thus serve as key limiting factors in natural HAB formation [20–23]. Dissolved inorganic nitrogen and phosphate (DIN and DIP) are routinely monitored as part of HAB mitigation efforts. The chemical forms of these substances are crucial to their bioavailability to phytoplankton [12, 23–31]. For example, orthophosphate (Pi) is the most bioavailable P source to phytoplankton, although its concentration in marine environments is typically much lower than that of dissolved organic phosphate [20, 23, 32–36]. While the machineries of utilization of dissolved phosphorous sources by phytoplankton are well characterized [23], a large part of marine P cycling, presumably driven by bacteria or archaea, remain to be better elucidated [37].

Several species of phytoplankton, including HAB species, are mixotrophic and capable of phagocytosing bacteria. Mixotrophy has been reported across multiple phyla, including dinoflagellates [38], haptophytes [39, 40], cryptophytes [41–44], and various green algae [39, 45]. Heterotrophy is often associated with nutrient-limiting conditions, such as environments deficient in Pi [46, 47], N [47], or dissolved organic carbon [47], or under dark conditions [47, 48], although some species exhibit consistent grazing behavior under various conditions [42]. Some species show a strong preference for live prey [39, 45, 49], while others are capable of ingesting dead bacteria or non-nutritive particulate matter [42–44, 46, 47, 49–51]. Mixotrophy encompasses a wide spectrum of lifestyles, ranging from obligate photosynthetic organisms that supplement their nutrition through phagocytosis, to predominantly heterotrophic organisms that rely on autotrophy when organic nutrients are scarce. Predominantly phototrophic phytoplankton may engage in bacterivory to supplement the limiting elements when needed. In addition, heterotrophy may allow these algae to utilize a wider range of organic compounds, including N- and P-containing substances that cannot be efficiently assimilated in a photoautotrophic state. These capabilities may enhance survival under Pi-limiting conditions, providing a competitive advantage over purely autotrophic ones.

To date, investigations on mixotrophy have mainly focused on the grazing activity of phytoplankton on prey—whether live or dead—under various conditions. However, studies examining the physiological consequences of nutrient uptake, specifically the propagation of the predator, remain relatively scarce. Furthermore, to the best of our knowledge, no studies have investigated the differential impacts of various prey species on the predator, such as differences in the degree of algal propagation induced by specific prey types.

*H. akashiwo* is a cosmopolitan species that frequently causes high-density blooms, sometimes resulting in massive fish kills. The organism expresses a high propagation rate, with a doubling time of approximately 24 h or less [52–54]. It is also allelopathic to several phytoplankton, *Akashiwo sanguia*, *Skeletonema costatum*, and *Thalassiosira rotula* [55–57], and is capable of utilizing both inorganic and organic phosphate [26, 58]. As *H. akashiwo* has been reported to be mixotrophic [59–61], we hypothesized that it may phagocytose bacteria to supplement its nutritional needs, and we searched for bacterial strains that support the growth of the alga under Pi-depleted conditions. Here, we demonstrate that *H. akashiwo* phagocytoses various bacterial species under Pi-depleted conditions and exhibits differential propagation depending on the prey species. We focus particularly on the bacterium prey *Vibrio comitans*, which promoted the highest level of algal propagation among the tested species, and we investigate the underlying mechanism in detail.

## Materials and methods

### Isolation of bacterial strains from environmental samples and externally provided bacterial strains

One or ten microliters of marine water collected from the locations listed in Table S1 were diluted to 100 μL in sterilized Daigo artificial seawater (ASW, Fujifilm Wako Chemicals, Osaka, Japan), spread on agar plates containing one-fifth strength commercial Difco™ Marine Broth 2216 (Becton Dickinson and Company, Franklin Lakes, NJ, USA) prepared in four-fifths strength ASW. Colonies were transferred to full-strength Difco™ Marine Broth 2216 plates at least twice to obtain isolated colonies. The 16S rRNA gene was amplified by colony polymerase chain reaction (PCR) and Sanger-sequenced [62], and the most homologous strains in the EZ-Taxon database were identified to determine the species [63, 64].

*V. comitans* strains NBRC1020676, −1 020 677, −1 020 679, −1 020 680, and −1 020 681 were obtained from the National Biological Resource Center at the National Institute of Technology and Evaluation (Tsukuba, Japan). *E. coli* strains MG1655 and MT4(pBC29) [65, 66] were provided by Dr. Kuroda (Hiroshima University, Japan).

### Culture conditions and co-culture experiments

The *H. akashiwo* H93616 strain (isolated from Uranouchi Bay, Kochi Prefecture, Japan) was used throughout this study. The strain was maintained in ASW supplemented with IMK (Fujifilm Wako Chemicals, Osaka, Japan) and antibiotics: penicillin (100 units/mL), streptomycin (100 μg/mL), ampicillin (100 μg/mL), and kanamycin (60 μg/mL). Cultures were maintained in a controlled-environment chamber with a 12 h light (100 μmol m^−2^ s^−1^)/12 h dark photoperiod at 29°C.

For co-culture experiments, algal cultures were diluted in antibiotic-free medium at <1/20, and maintained for one week before experiments. The refreshed *H. akashiwo* culture was preconditioned by diluting it to one-fifth volume in the IMK medium without phosphate (custom formulated by Nacalai Tesque, Kyoto Japan, IMK-PO_4_^3−^) and maintained for two days, followed by a second one-fifth dilution in IMK-PO_4_^3−^ and incubated for another two days. In all experiments, the propagation of *H. akashiwo* ceased after this final two day incubation, suggesting that the intracellular phosphate reservoir was depleted.

After the precondition steps, the concentration of Pi in the culture was as low as 1/25 of that in standard IMK medium, or 1.6 μM, while *H. akashiwo* densities exceeded >2 × 10^4^ cells/mL in all experiments. Bacterial strains were grown in Difco™ Marine Broth 2216 to OD_600_ > 1, rinsed twice in sterilized ASW, and adjusted to OD_600_ = 0.2 in sterilized ASW. Note that ASW is Pi-free; thus, the bacterial suspensions did not contain Pi.

For co-culture setup, 60 μL of bacterial suspension and 6 mL of *H. akashiwo* culture (adjusted to 200 cells/mL in IMK-PO_4_^3−^) were combined in non-treated 6-well culture plates (Iwaki; AGC Techno Glass Co. Ltd., Shizuoka, Japan). Because the preculture medium was maintained at 1/25-strength IMK and the culture was adjusted to 200 cells/mL for most experiments, corresponding to approximately 1/100 of the preculture in IMK-PO_4_^3−^, the Pi concentration in the starting co-culture contains <1/2500 of PO_4_^3−^ of that in standard IMK, corresponding to <16 nM. For co-culture experiments maintained in continuous darkness, the initial *H. akashiwo* density was adjusted to 1000 cells/mL. *H. akashiwo* cells were counted using a Moxi Z cell counter (ORFLO Technologies, Hailey, ID, USA) at the indicated timing.

### Bacterivory analysis

Bacterial strains were prepared as described in *culture conditions and co-culture experiments*. A fluorescent dye Cell Tracker™ Green CMFDA (5-chloromethylfluorescein diacetate, Thermo Fisher Scientific, Waltham, MA, USA), which specifically stains viable bacterial cells, was added to a final concentration of 10 μM in 1 mL of bacterial suspension. The mixtures were incubated for 30 min at room temperature. The cells were then washed twice in ASW, resuspended in 100 μL, and 5 μL of the suspension of stained cells was added to 100 μL of *H. akashiwo* culture.

After incubation under fluorescent light, the *H. akashiwo* and fluorescent bacteria mixtures were analyzed using a Moxi Go II Mini Automated Cell analyzer (ORFLO Technologies, Hailey, ID, USA) equipped with a 488 nm laser and 525/45 nm and 561 nm/LP filters, or observed using an FV1000 confocal microscope (Olympus, Tokyo, Japan).

### Genome sequencing of *Vibrio* strains

Genomic DNA of *V. comitans* Tj87, *V. rotiferianus Sk94*, and *V. owensii* Sk93 were purified using a DNeasy Blood & Tissue Kit (Qiagen, Germantown, MD). The extracted DNA was processed in Genome Lead Co. (Takamatsu, Japan) for DNA sequencing on the MiSeq platform for all *Vibrio* strains, and on a Nanopore platform for *V. comitans*. The adaptor sequences of Illumina reads were removed, and reads were quality-trimmed using fastp [67]. Reads from *V. rotiferianus* and *V. owensii* were assembled using Platanus B software [68], while reads for *V. comitans* were assembled using wtdbg2 [69]. Gene prediction and annotation were carried out using the D-FAST system (https://dfast.ddbj.nig.ac.jp), and the genome sequences and annotation was deposited to Database DNA Data Bank of Japan/NCBI, under accession numbers BAAIIC010000001 (*V. comitans* Tj87), BAAIID010000001–BAAIID010000182 (*V. owensii* Sk93), and BAAIIE010000001–BAAIIE010000287 (*V. rotferianus* Sk94). The identified polyphosphate kinases (PPKs) as well as PPKs sequences obtained from NCBI database (Appendix SI) were subjected to the phylogenetic analyses.

Phylogenetic analyses were conducted using GenomeNet (https://www.genome.jp/tools-bin/ete). Alignment and phylogenetic reconstructions were performed using the “build” function of ETE3 3.1.3 [70]. Alignment was performed with MAFFT v6.861b using the default settings [71]. The best proteins model was selected from JTT, WAG, VT, LG, and MtREV models with fixed parameters (+G + I + F) using ProtTest [72], as implemented in pmodeltest v1.4. A maximum likelihood tree was inferred using PhyML v20160115 with the following parameters: —pinv e —alpha e —nclasses 4 -o tlr -f m—bootstrap 100 [73]. Branch supports were calculated from 100 bootstrapped trees.

### polyP extraction and quantification

polyP extraction and quantification were carried out with minor modifications to previously published protocols [74, 75]. Briefly, cultured *Vibrio* cells were collected by brief centrifugation. To remove external calcium and various ions, the cells were rinsed twice with 0.4 M mannitol, the supernatant was carefully removed, and the cell pellet was snap-frozen and stored at −80°C until polyP extraction. PolyP was extracted using a NucleoSpin Gel and PCR Clean-up XS (MACHEREY-NAGEL GmbH & Co., Duren, Germany) according to manufacturer’s instructions with modified buffers as previously described [74].

polyP concentration was measured based on the amount of adenosine triphosphate (ATP) generated from polyP in the sample via a reaction with PPK in the presence of adenosine diphosphate (ADP), which corresponds to the total number of Pi residues in the polymer [75]. ATP was quantified using an ATP Bioluminescence assay kit (Roche, Mannheim, Germany) according to the manufacturer’s protocol. Protein concentrations were measured using a Bradford protein assay kit (BIO RAD, Hercules, CA, USA) with bovine serum albumin as the standard, according to the manufacturer’s protocol.

## Results

### Variety of bacterial species promote the growth of *H. akashiwo* under orthophosphate-depleted conditions

**Figure 1:**
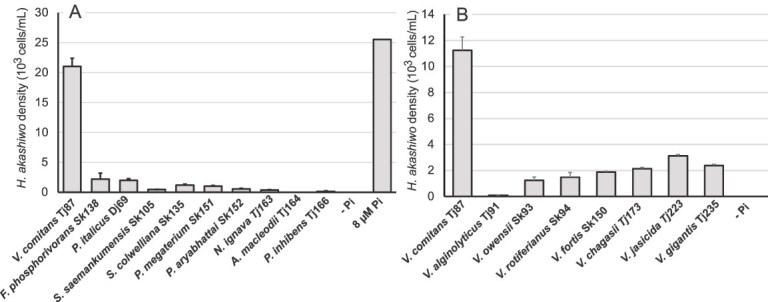
(A) *H. akashiwo* propagation with ten bacterial species under pi-depleted conditions. *H. akashiwo* adjusted to 200 cells/mL were inoculatd with or without bacteria under Pi-depleted conditions and the algal densities were measured at 12 days post-inoculation (dpi). As a positive control, the cell number of the alga cultured with 8 μM PO_4_^3−^ is presented. The Pi concentration in the *H. akashiwo* culture used for co-culture experiments was <1/2500 of the IMK medium, or 16 nM, and its nominal effect on *H. akashiwo* propagation was confirmed (-PO_4_^3−^). (B) *H. akashiwo* propagation, initially inoculated at 200 cells/mL, with *V. comitans* and seven other *Vibrio* species under Pi-depleted conditions at 11 dpi. Data are presented as the mean ± standard deviation of triplicate cultures measured twice. Note that the results shown in panel A and B are from independent experiments.

A total of 10 bacterial strains isolated from Japanese coastal waters were tested for their ability to support the growth of *H. akashiwo* under Pi-depleted conditions (Fig. 1A). Importantly, eight strains, including *Fictobacillas phosphovorans* strain Sk138, *Phaeobacter italicus* strain Dj69, *V. comitans* strain Tj87, *Seohaeicola saemankumensis* strain Sk105, *Shewanella colwelliana* strain Sk135, *Priestia magaterium* strain Sk151, *Priestia aryabhattai* strain Sk152, and *Nereida ignava* strain Tj163, markedly promoted the proliferation of *H. akashiwo*, with cell densities increasing by tenfold or more from the initial concentration (200 cells/mL). Among these, *V. comitans* strain Tj87 supported the propagation of *H. akashiwo* to >2 × 10^4^ cells/mL.

In contrast, *H. akashiwo* cultures supplemented with *P. inhibens* strain Tj166 remained at nearly constant density, and the algal culture supplemented with *A. macleodii* strain Tj164 declined to extinction (Fig. 1A). Because the Pi concentration in the *H. akashiwo* culture was <1/2500 of the phosphate level in IMK medium, which is as low as <16 nM, and because bacterial suspensions were washed twice and resuspended in Pi-free ASW (see Materials and methods), the primary P source in the co-culture system, if any, must be the added bacteria.

Next, we examined whether the growth-promoting effect observed under these conditions is common among species of the *Vibrio* genus (Fig. 1B). Seven *Vibrio* species, including *V. comitans* strain Tj87, *V. owensii* strain Sk93, *V. rotiferianus* strain Sk94, *V. fortis* strain Sk150, *V. chagasii* strain Tj173, *V. jasicida* strain Tj223, and *V. gigantis* strain Tj235, promoted the growth of *H. akashiwo*, although the magnitude of the effect varied widely. In contrast, the cell density of *H. akashiwo* supplemented with *V. alginolyticus* strain Tj91 remained stable throughout the experiment (Fig. 1B).

Taken together, several of the bacteria tested here exhibited a growth-promoting effect on Pi-depleted *H. akashiwo*, with *V. comitans* showing a particularly strong influence, comparable to the effect of ~8 μM Pi (Fig. 1A), which corresponds to eutrophic conditions in nature. Furthermore, co-culture experiments using five additional *V. comitans* strains obtained from the National Biological Resource Center (NBRC, Tsukuba, Japan) confirmed that this bacterial species promotes the proliferation of *H. akashiwo* under Pi-depleted conditions (Fig. S1).

### *H. akashiwo* phagocytose *V. comitans* under orthophosphate-depleted and -replete conditions

**Figure 2:**
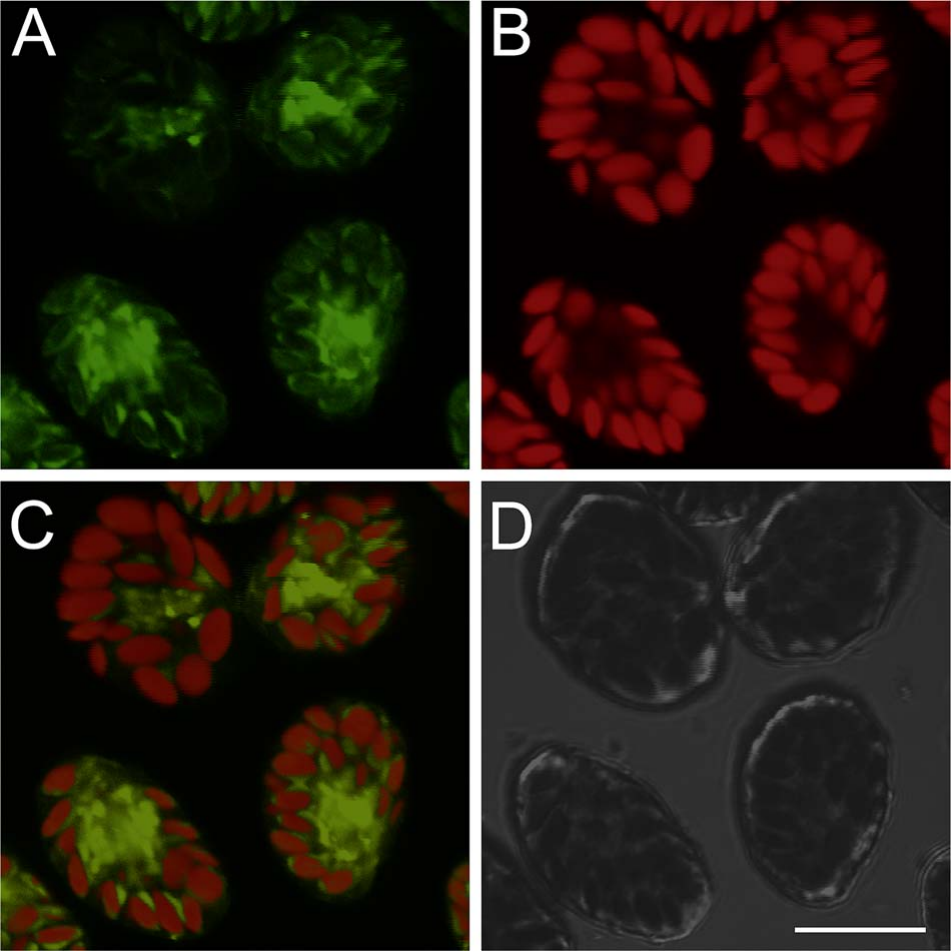
*H. akashiwo* internalized *V. comitans. V. comitans* was pre-stained with CellTracker™, then incubated with *H. akashiwo* for 30 min. The alga was visualized under a confocal microscope. (A) Channel for *V. comitans* stained with CellTracker™, (B) autofluorescence of chloroplasts, (C) signal from *V. comitans* overlaid on the chloroplast autofluoresence, (D) bright-field image. Bar = 10 μm.

As *H. akashiwo* is reported to be bacterivorous [59–61], we next examined whether *V. comitans* is phagocytosed by the alga. Live *V. comitans* cells were stained by a fluorescence dye Cell Tracker™ (5-chloromethylfluorescein diacetate, Thermo Fisher Scientific, Waltham, MA, USA), and the culture of *H. akashiwo* supplemented with dye-labeled bacterial cells was imaged using confocal laser scanning microscopy (Fig. 2). *H. akashiwo* exhibits strong red autofluorescence due to its chloroplasts (Fig. 2B). An optical section near the center of an *H. akashiwo* cell revealed a condensed green signal within the space surrounded by chloroplasts aligned along the cell surface (Fig. 2A–C). On contrary, *H. akashiwo* cells without fluorescently-labeled bacterium did not exhibit such autofluorescence (Fig. S2). These data collectively indicate that *V. comitans* was internalized by the phytoplankton.

**Figure 3:**
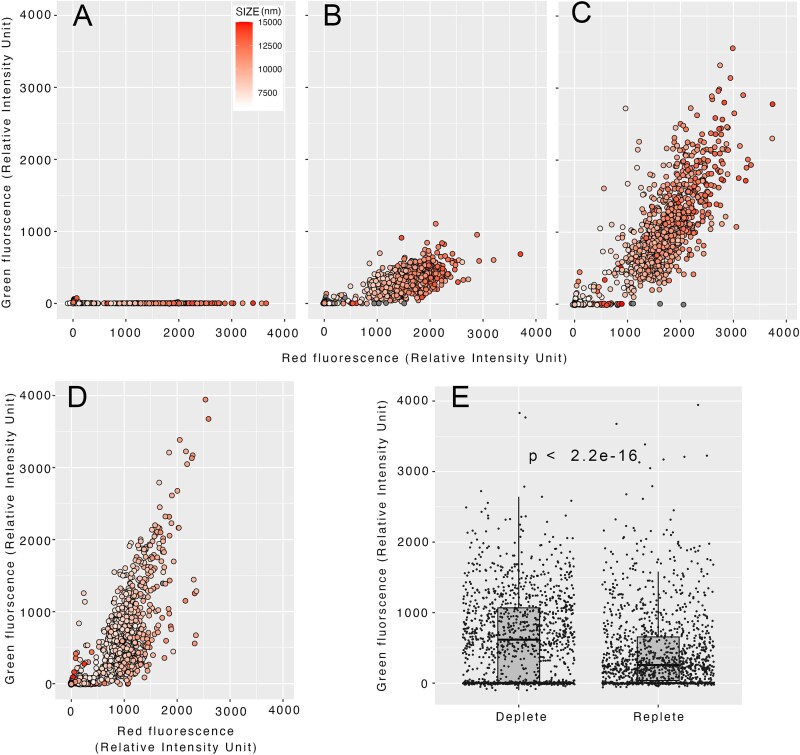
Cytograms of *H. akashiwo* cultured in P-depleted (A–C) and -replete (D) media and inoculated with *V. comitans*, taken at 0 (A), 30 (B), and 60 min (C, D) after the addition of Cell tracker™-stained *V. comitans*. CellTracker™- and autofluorescence signals were derived from labeled *V. comitans* and the chloroplasts of *H. akashiwo*, respectively. The color intensity of the dots indicates the size (>6 μm diameter) of the analyzed particles correspond to cell size of the alga, and the gray dots represent the particles smaller than the cutoff. (E) Comparison of the intensities of CellTracker™-signals associated with *H. akashiwo* in P-depleted or -replete medium at 60 min after the co-incubation started. The significance of the difference between the results was analyzed by Welch’s two-sample *t*-test.

This bacterivory was further confirmed using flowcytometry. Following the addition of green fluorescence–labeled *V. comitans*, the intensity of the green signal associated with red-fluorescent particles greater than 6 μm in diameter, which corresponds to algal cells, markedly increased in a time-dependent manner (Fig. 3A–C). Although green signals were also detected in Pi-replete *H. akashiwo* (Fig. 3D), their intensity was significantly lower compared to those under Pi-depleted conditions (Fig. 3E), suggesting that phagocytosis may be promoted by Pi depletion though nutritionally replete alga still ingest bacteria to a significant extent.

To assess whether *H. akashiwo* exclusively phagocytoses *V. comitans*, we performed the same experiments using *V. alginolyticus*, a species that did not promote algal growth (Fig. 1B). *V. alginolyticus* was also ingested by the phytoplankton to a similar extent as *V. comitans* in a time-dependent manner (Fig. S3).

### Growth promotion effect of *V. comitans* on *H. akashiwo* requires light

**Figure 4:**
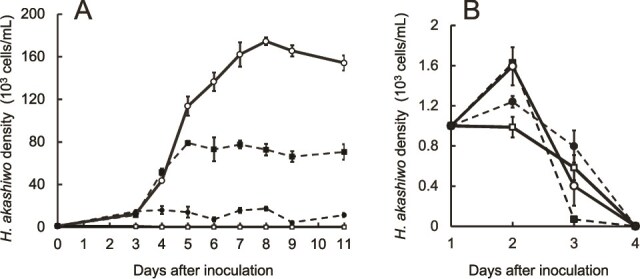
Densities of *H. akashiwo* cultures with or without *vibrio* strains under PO_4_^3—^depleted conditions in 12 h light: 12 h dark cycles (A) and continuous darkness (B). *V. comitans* (open circles with solid line), *V. rotiferianus* (closed squares with dashed line), and *V. owensii* (closed circles with dashed line) that were pre-cultured in Difco™ Marine Broth 2216 were added to the *H. akashiwo* culture maintained in PO_4_^3-^depleted conditions. Densities of *H. akashiwo* cultured in the medium without the addition of bacteria (open squares and solid line) were also measured. Initial *H. akashiwo* densities were adjusted to 200 cells/mL (A) and 1000 cells/mL (B). Data are presented as the mean ± standard deviation of triplicate cultures measured twice.

Next, we examined whether *H. akashiwo* proliferation in response to the addition of *V. comitans* under Pi-depleted conditions required algal photosynthesis. Algal growth was only observed when the culture with *V. comitans* was maintained under a light: dark cycle (Fig. 4A). When the culture was kept in continuous darkness, the *H. akashiwo* density showed a slight increase over the first two days, then declined and eventually reached extinction (Fig. 4B).

### Polyphosphate accumulated in *V. comitans* serves as a orthophosphate source for *H. akashiwo*

**Figure 5:**
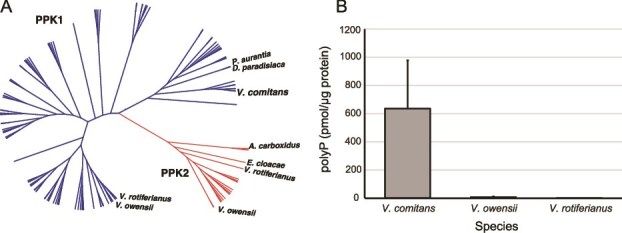
(A) Phylogeny of PPKs in *Vibrio* species. (B) polyP contents in *V. comitans*, *V. rotiferianus*, and *V. owensii*. Data are presented as the mean ± standard deviation of quadruplicate samples.

To identify the P compound(s) that could substitute for soluble Pi in the medium, we obtained the complete genome sequence of *V. comitans* Tj87 and draft genome sequences of *V. rotiferianus* and *V. owensii* strains. We found that all three strains possess polyphosphate (polyP) synthesizing genes, known as PPKs. Bacterial PPKs are classified into two types: PPK1 and PPK2 [76]. Previous studies have suggested that PPK1 primarily synthesizes polyP, while PPK2 catalyzes both the addition and removal of phosphate from the substrate [76]. Genome sequence analyses revealed that *V. comitans* possesses only PPK1, whereas *V. rotiferianus* and *V. owensii* possess both PPK1 and PPK2 (Fig. 5A).

Next, we quantified the polyP accumulation in these *Vibrio* strains. Only *V. comitans* accumulated detectable levels of polyP under the experimental conditions (Fig. 5B). Microorganisms are known to store polyP as long chains composed of 100 to 1000 phosphate units within storage granules known as acidocalcisomes [37]. In *V. comitans*, we observed similar granule-like structures, and energy-dispersive X-ray spectroscopy confirmed the P enrichment within these structures (Fig. S4A–B).

**Figure 6:**
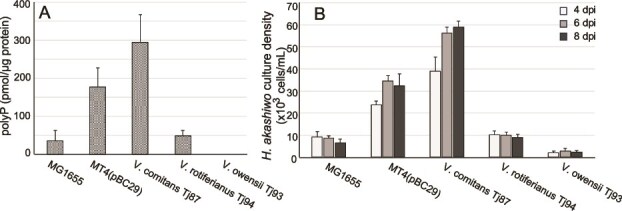
polyP content (A) and propagation of *H. akashiwo* (B) co-cultured *E. coli* MG1655, polyP-accumulating *E. coli* strain MT4(pBC29), *V. comitans* Tj87, *V. rotiferianus* Tj94, and *V. owensii* Tj93. Data are presented as the mean ± standard deviation of triplicate cultures measured twice.

To further confirm that polyP in bacteria serves as a Pi source for the alga, we attempted to generate a ∆*ppk1* strain of *V. comitans*, but were unsuccessful. As an alternative approach, we conducted a co-culture experiment of *H. akashiwo* and *E. coli* strain MT4(pBC29), which is genetically engineered to accumulate polyP at high levels compared to its wild-type counterpart, MG1655 [65, 66]. The MT4(pBC29) strain accumulated polyP at approximately 60% of the levels observed in *V. comitans* (Fig. 6A). In the co-culture, *H. akashiwo* exhibited 61% of the growth observed when co-cultured with *V. comitans* (Fig. 6B).

## Discussion

Bloom-forming algae predominate in local populations due to their fast growth and ability to accumulate at high densities. Efficient nutrient acquisition is presumably fundamental to support their propagation. Here, we found that the bloom-forming species *H. akashiwo*, which sometimes forms dense blooms in the environment, is capable of ingesting bacteria and exploiting polyP as a nutrient—and probably as an energy source as well—to conduct photosynthesis.

The growth promotion of *H. akashiwo* by bacterial strains under Pi-depleted conditions was relatively common, although the extent varied widely depending on the strain tested (Fig. 1). Among them, *V. comitans* promoted the proliferation of *H. akashiwo* to a prominent degree (Fig. 1B, Fig. S1), while the phytoplankton also ingested other *Vibrio* species that did not support algal propagation (Fig. S2). The propagation of *H. akashiwo* upon bacterivory may occur purely through heterotrophic metabolism, by utilizing organic P-containing substances such as ATP, nucleotides, or phospholipids derived from the phagocytosed bacteria. Alternatively, *H. akashiwo* may assimilate bacterium-derived P-containing substances during photosynthesis to support propagation. Since *H. akashiwo* co-cultured with *V. comitans* did not proliferate under constant darkness, it suggests that the alga assimilates *V. comitans*–derived P-containing substances through photosynthesis (Fig. 4).

A study of the obligate phototrophic species *Ochromonas* spp. strain CCMP1393 showed enhanced growth in the presence of heat-killed bacteria under light [43]. However, in that case, the phytoplankton was maintained under nutritionally replete conditions (e.g. 36 μM PO_4_), obscuring the contribution of heterotrophic nutrition to growth [43]. The heat-killed bacteria likely supplied auxiliary factors that promoted propagation rather than essential nutrients for photosynthesis. In contrast, *H. akashiwo* propagates with *V. comitans* under Pi-depleted conditions in this study, indicating that the ingested bacterium likely provides otherwise deficient phosphorous, or more specifically Pi, for photosynthesis.

Is there a P-containing compound uniquely present in *V. comitans* that promotes the growth of *H. akashiwo* to a much greater extent than other species? One candidate is the linear polymer of Pi, polyphosphate [77]. polyP is a highly anionic polymer composed of Pi units connected by high-energy phosphoanhydride bonds. Indeed, *V. comitans* accumulated polyP under the experimental conditions (Fig. 5B), while genomic analyses of *V. comitans*, *V. rotiferianus*, and *V. owenssi* revealed that all three species possess genes encoding PPKs, enzymes responsible for synthesizing polyP. Previous studies suggest that PPK1 primarily synthesizes polyP, whereas PPK2 catalyzes both the addition and removal of phosphate groups from the substrate [76]. The polyP levels observed in Fig. 5B are consistent with the absence of PPK2 in *V. comitans*. Because a exopolyphosphatase, which hydrolyzes polyP, was found in each of the *Vibrio* species, this enzyme may not be critical factor for the differential levels of polyP in these bacteria.

*V. comitans* and the strains tested in this study are heterotrophic bacteria. *V. comitans*, particularly, synthesize polyP by PPK1 from intracellular ATP yielded by the assimilation of organic matters. On the contrary, *H. akashiwo* directly assimilates Pi through photosynthesis. Because of the differential trophic orientations of these organisms, under marine environment with high organic matters with low Pi, *V. comitans* can proliferate and may accumulate polyP, while *H. akashiwo* suffers from low nutrition. In this study, such an interaction was simulated by culturing bacteria in rich medium and co-cultured with Pi-depleted *H. akashiwo* after rinsing the bacteria in Pi-free ASW. The observations obtained in this study provide novel scenario that bacteria proliferate on naturally occurring organic matter to accumulate polyP and subsequently phagocytosed and utilized by the alga in Pi-limited marine environment. Enhanced bacterivory by *H. akashiwo* under Pi-depletion (Fig. 3E) suggest that this process may take place even more efficiently under Pi-limited environment that previously believe to be limiting for *H. akashiwo* growth.

*H. akashiwo* significantly proliferated when co-cultured with the polyP-accumulating *E. coli* strain MT4(pBC29), further supporting that polyP-accumulating bacteria serve as efficient P sources (Fig. 6). The close correlation between polyP accumulation levels in various bacterial strains and *H. akashiwo* propagation extent demonstrates that bacterial polyP is the Pi source utilized by *H. akashiwo* under Pi-limiting conditions (Fig. 6). These data also suggest that *H. akashiwo* utilizes polyP-accumulating bacteria as a Pi source, regardless of bacterial species.

This is the first study to reveal that *H. akashiwo* phagocytoses bacteria containing polyP and utilizes it as a Pi source. On the other hand, *H. akashiwo* is known to store polyP intracellularly, presumably to adapt to Pi-depleted conditions [16, 78]. While the regulatory mechanisms governing intracellular polyP in phytoplankton remain enigmatic, it has been observed that polyP accumulates to high levels under prolonged Pi-depleted conditions, suggesting that this may be the appropriate timing for its utilization [78]. *H. akashiwo* is likely equipped with a polyP-utilizing system, enabling access to both Pi and the energy released from its hydrolysis [16]. In addition, utilization of polyP supplied exogenously in the medium by *H. akashiwo* has also been documented [16]; however, it remains unclear whether the alga takes up intact polyP or assimilates Pi released by hydrolysis of the polymer by polyP-digesting enzymes secreted by the organism. Since the *H. akashiwo* genome sequence is not yet available, the polyP-digesting enzymes and other components required for intracellular polyP utilization remain to be identified. With cellular machinery for phagocytosis, the entire polyP utilization pathway in the alga should be characterized in future studies.

It is noteworthy that *H. akashiwo* co-cultured with *V. comitans* in the presence of 8 μM Pi propagated to a higher density than the alga cultured without bacteria (Fig. S5). These results aligns with the observation that *H. akashiwo* phagocytoses *V. comitans* under Pi-replete conditions, although at a lower rate than under Pi-depleted conditions (Fig. 3E). These results collectively indicate that *H. akashiwo* can utilize Pi sources in both soluble and particulate forms in a cumulative manner. This dual-mode nutrient uptake presumably occurs under natural conditions and provides additional competitiveness to the species.

Because *Vibrio* species other than *V. comitans* and some other bacterial strains showed a much lower but definite growth-promoting effect on *H. akashiwo* while accumulating negligible levels of polyP, other substance(s) contained in these bacterial strains may also serve as P source(s) for the alga. Because several molecular species of dissolved organic phosphate were demonstrated to be utilized by the alga, such components can be assimilated upon the phagocytosis of these bacteria [23, 26]. Such P acquisition may be taken place in the marine environment and support the phytoplankton community even at the minor extent.

Our results here propose a “hybrid” trophic strategy of *H. akashiwo*, funneling the nutrients obtained through heterotrophic bacterivory as well as soluble, inorganic nutrition sources into the photosynthesis pathway. Metatranscriptomic analysis of *H. akashiwo* blooms and laboratory cultures demonstrated upregulation genes related to dissolved organic phosphate uptake and mixotrophy during sharp declines of ambient Pi [15, 16, 43], supporting the involvement of *H. akashiwo* bacterivory during bloom formation.

This study provides proof of concept for the importance of microbial flora composition in influencing the likelihood of bloom occurrence. The presence of polyP-containing marine bacteria, as well as the distribution of bacterivory-competent phytoplankton species, may be important factors shaping local ecosystems and bloom potential. The possibility for other bloom-forming species to utilize bacteria-derived nutrition through bacterivory should also be investigated in future studies. Further, the role of algal bacterivory–mediated P incorporation in primary production and total P cycling in ecosystems warrants further evaluation. The level of polyP in PPK-harboring bacteria may be determined by various environmental condition, and this may affect the primary production level of the phytoplankton community achieved through the phagocytosis-mediated polyP uptake.

Finally, this study highlights a potential limitation of conventional DIP monitoring for understanding HAB dynamics. Our results strongly suggest that, in addition to dissolved inorganic and organic P-containing substances, the microbial population, specifically, the total amount of polyP accumulated in the microbial flora, may significantly influence the algal population in the environment. Thus, the microbiota composition, including algae with diverse trophic modes and bacteria rich in polyP or other P-containing molecules, may be crucial determinants of HAB occurrence.

## Supplementary Material

SFIg1_ycaf192

Sfig2_new_ycaf192

SFig3_new_ycaf192

SFig4_new_ycaf192

SFig5_new_ycaf192

TableS1_062425_ycaf192

SI_Appendix_ycaf192

SuppMM_062425_ycaf192

## Data Availability

All data generated or analysed during this study are included in this published article and its supplementary information files.
