# Supplementary material for: Proliferation of a bloom-forming phytoplankton via uptake of polyphosphate-accumulating bacteria under phosphate-limiting conditions
Source: ISME Commun. 2025 Dec 5;5(1):ycaf192. doi: 10.1093/ismeco/ycaf192 (PMC12684721; doi:10.1093/ismeco/ycaf192)
Supplement: SFIg1_ycaf192 [file sfig1_ycaf192.pdf]

Supplemental Figure 1

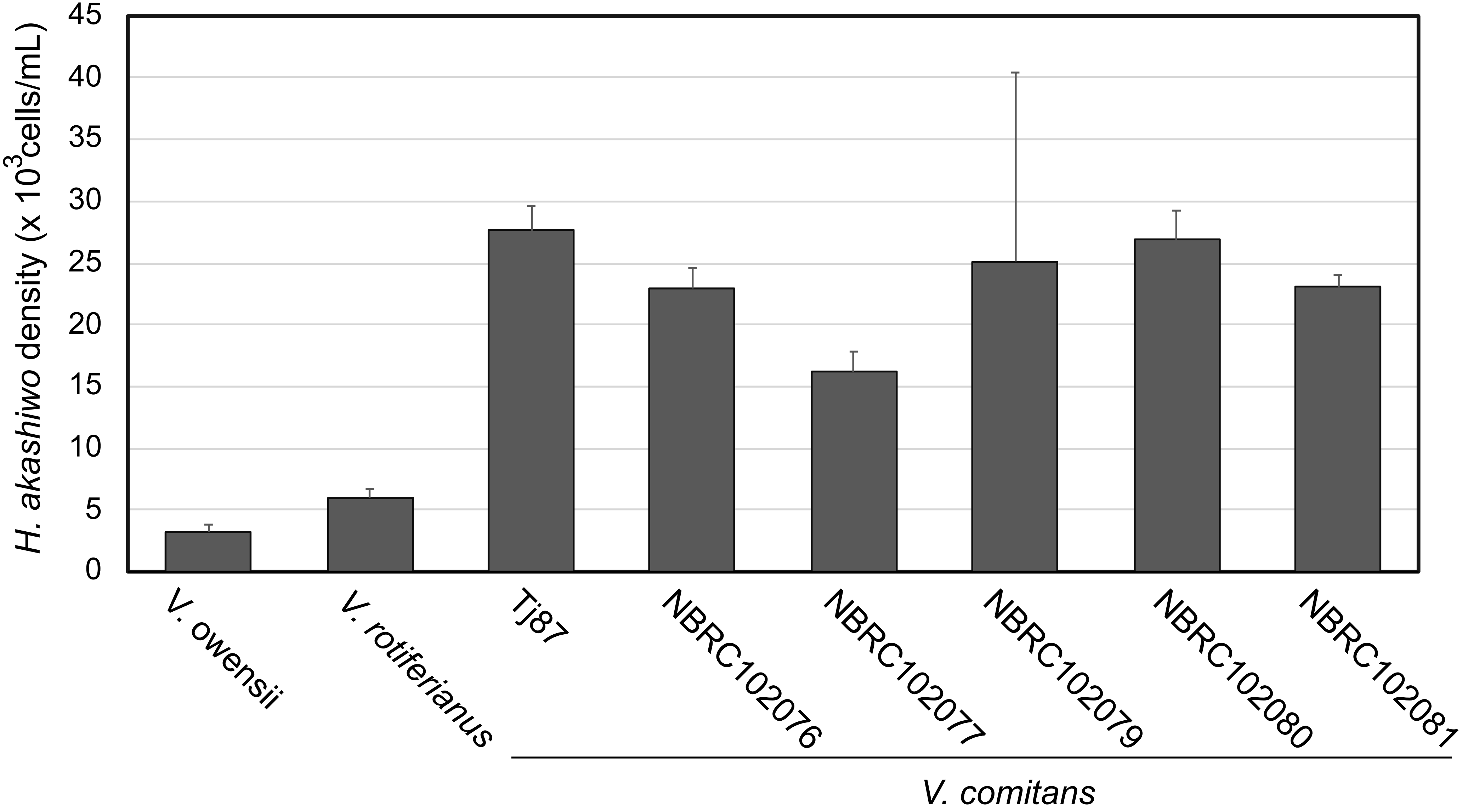

SFig. 1 *H. akashiwo* propagation with *V. owensii*, *V. rotiferianus*, and six *V. comitans* strains under Pi-depleted conditions. Cell numbers of *H. akashiwo* cultured with or without bacteria were measured at 12 dpi. Data are presented as the mean ± standard deviation of triplicate cultures measured twice.
