# Supplementary material for: Proliferation of a bloom-forming phytoplankton via uptake of polyphosphate-accumulating bacteria under phosphate-limiting conditions
Source: ISME Commun. 2025 Dec 5;5(1):ycaf192. doi: 10.1093/ismeco/ycaf192 (PMC12684721; doi:10.1093/ismeco/ycaf192)
Supplement: Sfig2_new_ycaf192 [file sfig2_new_ycaf192.pdf]

Supplemental Figure 2

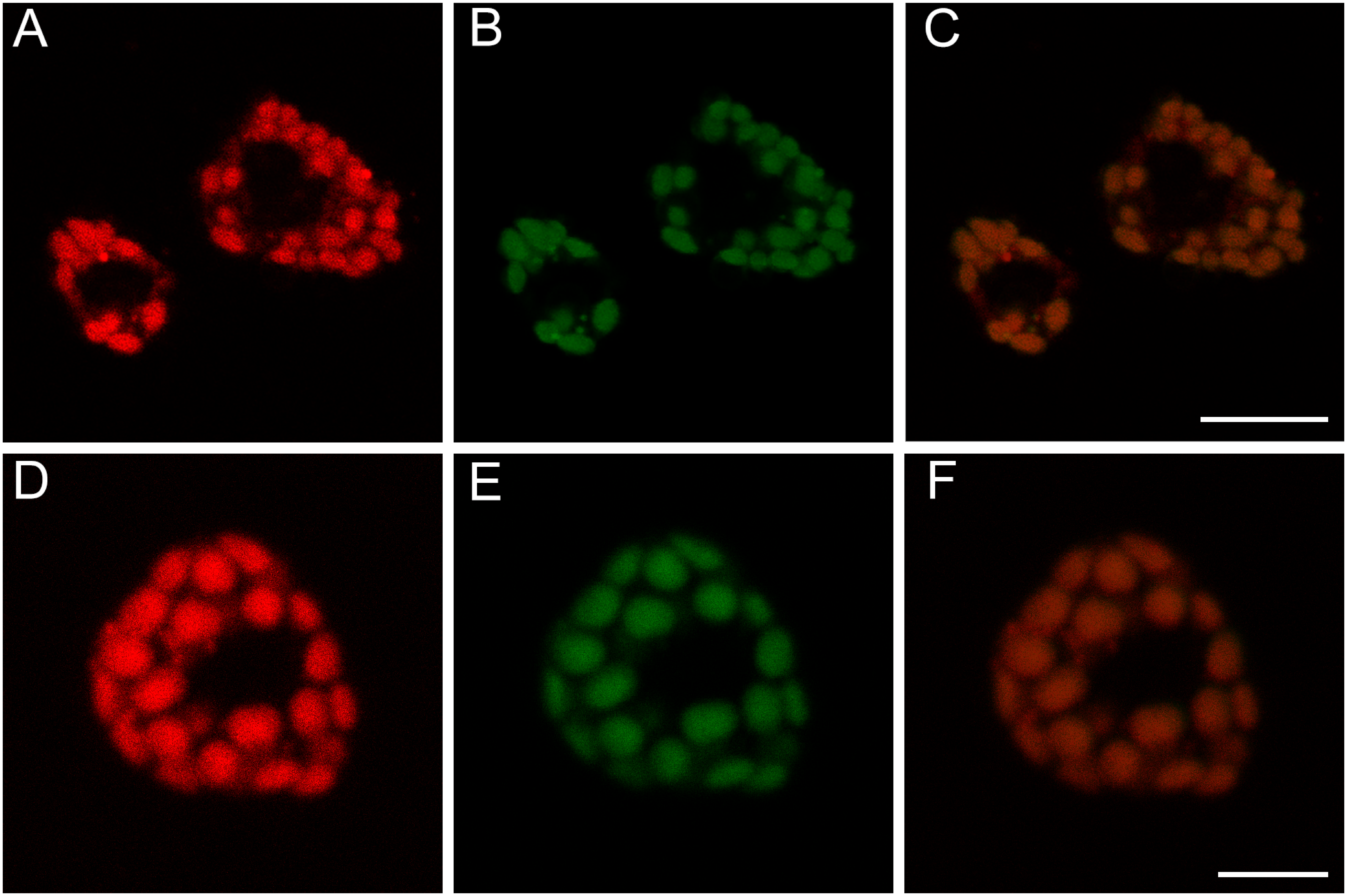

SFig 2 Representative images of *H. akashiwo* visualized under a confocal microscope to confirm the absence of autofluorescence detected in green channel. (A, D) Green channel, (B, E) autofluorescence of chloroplasts, (C, F) green signal overlaid on the red signal, Bars = 10 μm. Note that the gain for the green channel was intensified to the level that faint autofluorescence from chlorophyll can be detected so that the absence of the signal resembling the phagocytosed stained bacteria observed in Fig 2 can be confirmed.
