# Supplementary material for: Proliferation of a bloom-forming phytoplankton via uptake of polyphosphate-accumulating bacteria under phosphate-limiting conditions
Source: ISME Commun. 2025 Dec 5;5(1):ycaf192. doi: 10.1093/ismeco/ycaf192 (PMC12684721; doi:10.1093/ismeco/ycaf192)
Supplement: SFig3_new_ycaf192 [file sfig3_new_ycaf192.pdf]

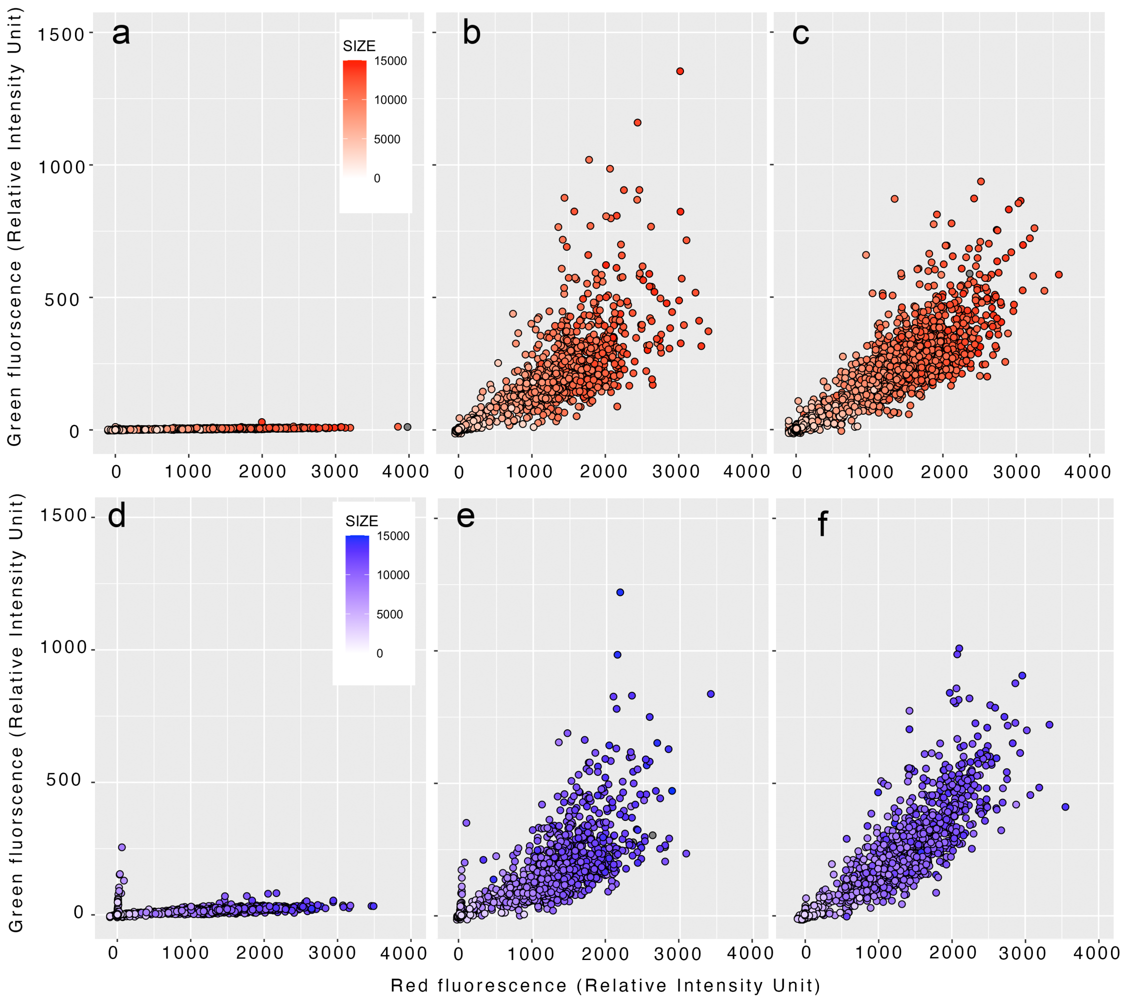

SFig. 3 Cytograms of *H. akashiwo* cultured in P-depleted medium and inoculated with *V. comitans* (a–c) or *V. alginlyticus* (d–f), taken at 0 (a, d), 30 (b, e), and 60 min (c, f) after the addition of Cell Tracker^{TM}–stained bacteria. Green and red fluorescence signals were derived from the labeled *V. comitans* and the autofluorescence of *H. akashiwo*, respectively. The unit for the size of the particles are nm, and the gray dots represents the particles with the diameter less than 6 µm
