# Supplementary material for: Proliferation of a bloom-forming phytoplankton via uptake of polyphosphate-accumulating bacteria under phosphate-limiting conditions
Source: ISME Commun. 2025 Dec 5;5(1):ycaf192. doi: 10.1093/ismeco/ycaf192 (PMC12684721; doi:10.1093/ismeco/ycaf192)
Supplement: SFig4_new_ycaf192 [file sfig4_new_ycaf192.pdf]

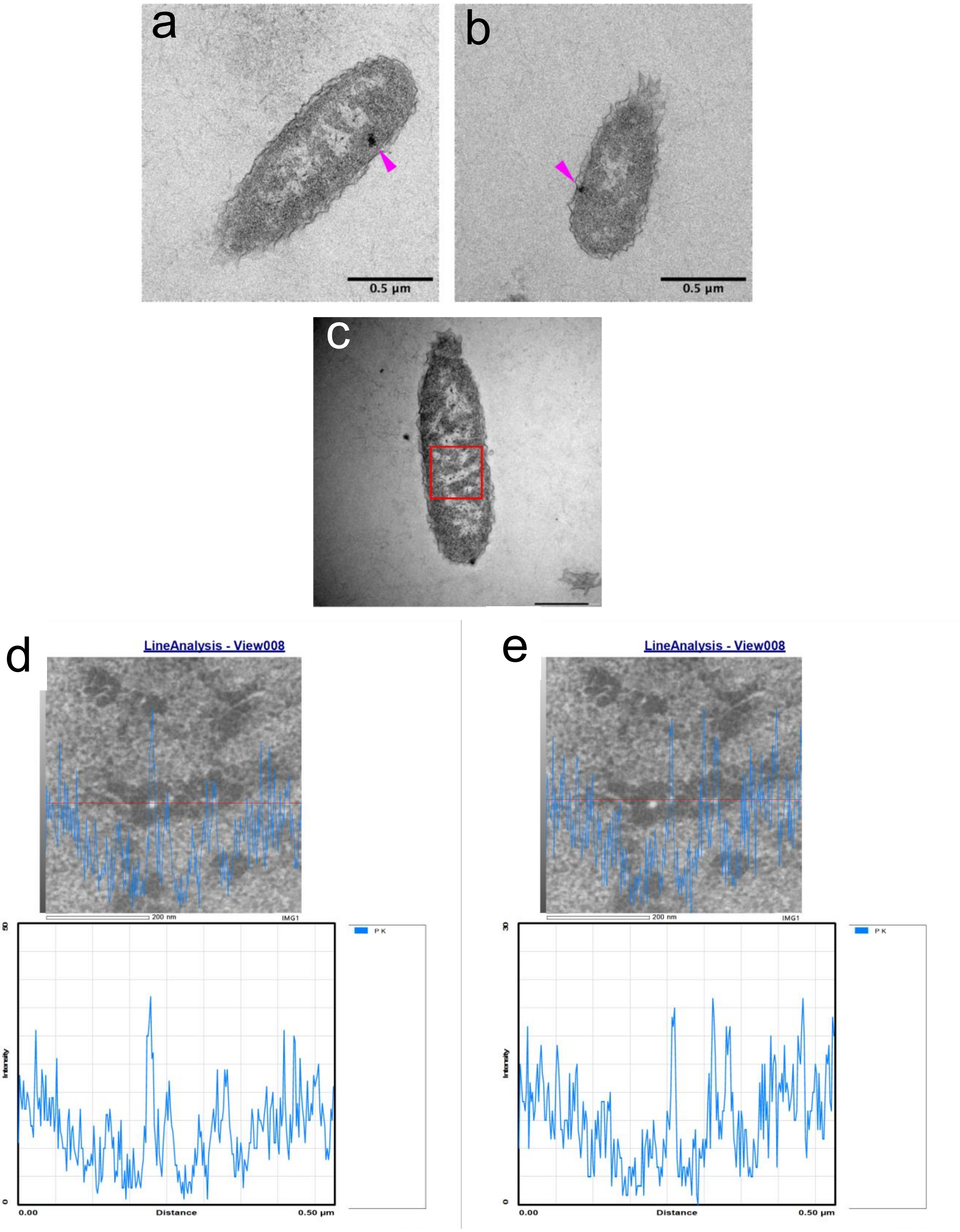

SFig. 4 Transmission electron micrographs of *V. comitans* sections (a, b, c). Black granules resembling previously reported polyP-containing acidocalcisomes are indicated by magenta arrowheads. The red square area in panel c was subjected to energy-dispersive X-ray spectroscopy to quantify the distribution of phosphorus and dark-field STEM images as backgrounds shown by the scale bars on the left side of each panel (d, e). The distribution of phosphorus along each horizontal red line was detected by EDX and visualized as superimposed blue lines and corresponding bottom charts.
