# Supplementary material for: Proliferation of a bloom-forming phytoplankton via uptake of polyphosphate-accumulating bacteria under phosphate-limiting conditions
Source: ISME Commun. 2025 Dec 5;5(1):ycaf192. doi: 10.1093/ismeco/ycaf192 (PMC12684721; doi:10.1093/ismeco/ycaf192)
Supplement: SFig5_new_ycaf192 [file sfig5_new_ycaf192.pdf]

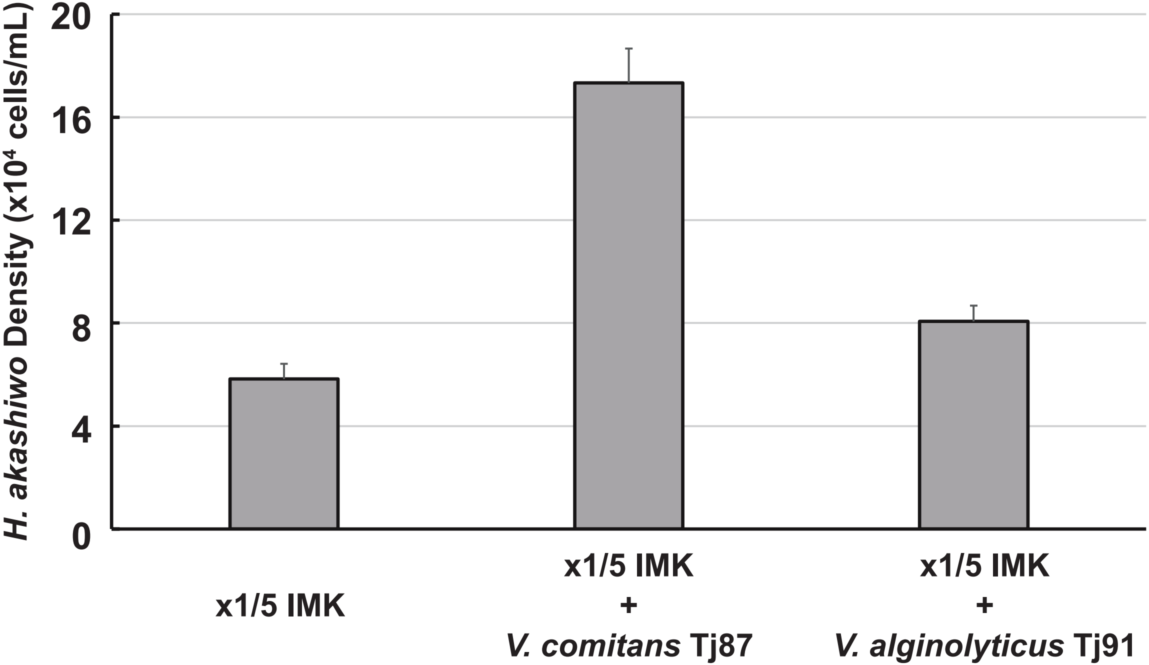

SFig. 5 *H. akashiwo* propagation with *V. comitans* Tj87 and *V. alginolyticus* Tj91 strains in sterilized artificial seawater supplemented with one-fifth strength IMK, which contains ~8 μM Pi. Cell numbers of *H. akashiwo* in each culture were measured at 12 dpi. Data are presented as the mean ± standard deviation of triplicate cultures measured twice.
