# Supplementary material for: Proliferation of a bloom-forming phytoplankton via uptake of polyphosphate-accumulating bacteria under phosphate-limiting conditions
Source: ISME Commun. 2025 Dec 5;5(1):ycaf192. doi: 10.1093/ismeco/ycaf192 (PMC12684721; doi:10.1093/ismeco/ycaf192)
Supplement: TableS1_062425_ycaf192 [file tables1_062425_ycaf192.docx]

STable 1 Bacterial strains isolated and analyzed for this study.

| Geological sources |  |  |
| --- | --- | --- |
| Species | Strain name | % identity* |
| Ieshima, Harima Bay, Hyogo Prefecture | |  |
| *Phaeobacter italicus* | Dj69 | 100.0 |
| Sakai Port, Sakai City, Osaka Prefecture | |  |
| *Fictibacillus phosphorivorans* | Sk138 | 99.0 |
| *Seohaeicola saemankumensis* | Sk105 | 99.7 |
| *Shewanella colwelliana* | Sk135 | 100.0 |
| *Priestia megaterium* | Sk151 | 100.0 |
| *Priestia aryabhattai* | Sk152 | 99.9 |
| *Vibrio owensii* | Sk93 | 100.0 |
| *Vibrio rotiferianus* | Sk94 | 99.9 |
| *Vibrio fortis* | Sk150 | 99.8 |
| Tajiri Port, Fukuyama City, Hirosima Prefecture | |  |
| *Vibrio comitans* | Tj87 | 100.0 |
| *Nereida ignava* | Tj163 | 100.0 |
| *Alteromonas macleodii* | Tj164 | 99.7 |
| *Phaeobacter inhibens* | Tj166 | 100.0 |
| *Vibrio alginolyticus* | Tj91 | 99.5 |
| *Vibrio chagasii* | Tj173 | 99.9 |
| *Vibrio jasicida* | Tj223 | 99.9 |
| *Vibrio gigantis* | Tj235 | 100.0 |
