# Supplementary material for: Proliferation of a bloom-forming phytoplankton via uptake of polyphosphate-accumulating bacteria under phosphate-limiting conditions
Source: ISME Commun. 2025 Dec 5;5(1):ycaf192. doi: 10.1093/ismeco/ycaf192 (PMC12684721; doi:10.1093/ismeco/ycaf192)
Supplement: SuppMM_062425_ycaf192 [file suppmm_062425_ycaf192.doc]

Supplemental Material: Experimental Procedure for SFig 3

Cells were fixed with 2.5% glutaraldehyde for 2 h at 4°C, washed three times with PBS (Fujifilm Wako Chemicals, Osaka, Japan) on ice, and post-fixed with 1% OsO_4_ for 2 h. They were then washed three times with MilliQ and dehydrated for 5 min in 30% ethanol, 10 min in 50% ethanol, 10 min in 70% ethanol, 10 min in 90% ethanol, 15 min in 95% ethanol, and 15 min in 100% ethanol twice. This was followed by propylene oxide substitution and EPON812 resin embedding (1:1 PO:EPON, 1:3 PO:EPON, then 100% EPON). Polymerization was conducted at 60°C for 2 days. Ultrathin sections (50 nm) were prepared using an ultramicrotome (UC7, Leica, Germany) and double-stained with 2% uranyl acetate and lead citrate. Sections were examined using transmission electron microscopes (Tecnai G2 Spirit, FEI Company, USA; JEM-F200, JEOL, Japan) at 120 kV or 200 kV and EDX measurement (EX-24390UBN5T dry SD100WL, JEOL, Japan).
